# Update on Uveitis Management

**DOI:** 10.1155/2015/382747

**Published:** 2015-02-23

**Authors:** Vishali Gupta, Quan Dong Nguyen, Manfred Zierhut, Ilknur Tugal-Tutkun

**Affiliations:** ^1^Advanced Eye Centre, Post Graduate Institute of Medical Education and Research, Chandigarh 160012, India; ^2^Stanley M. Truhlsen Eye Institute, University of Nebraska Medical Center, Omaha, NE 68198, USA; ^3^Center of Ophthalmology, University of Tuebingen, Tuebingen, Germany; ^4^Istanbul Faculty of Medicine, Istanbul University, Fatih, 34093 Istanbul, Turkey

Uveitis management has made a dramatic progress in the last decade with the development of new drugs as well as the change in the approach to the patients. There are several new drugs available including biologics, recombinant monoclonal antibodies against interleukins including IL-17, IL-l, and IL-6, and anti-TNF-*α* and T-cell inhibitors such as fusion proteins. In addition, there has been a shift in the drug administration with the preference of local administration of therapy by using intravitreal injection or iontophoresis. The widespread use of anti-VegF agents has brought a change in the management strategies of inflammatory choroidal neovascular membranes as well as uveitic macular edema. The availability of newer imaging techniques including fundus autofluorescence (FAF) for imaging of the retinal pigment epithelium and enhanced depth imaging of the choroid by OCT too has influenced the management of uveitis by titrating the therapy for each patient.

This special issue on update in uveitis management has review articles as well as original articles addressing these issues. In a review article, “Role of Autofluorescence in Inflammatory/Infective Diseases of the Retina and Choroid,” A. Samy et al. have reviewed the published literature on FAF in inflammation of the posterior segment, specifically patterns in infectious and noninfectious uveitis, and illustrated their relevance with the help of illustrations and case histories. The review by A. Baltmr et al. “Examining the Choroid in Ocular Inflammation: A Focus on Enhanced Depth Imaging” summarizes the current application of EDI technique in ocular inflammatory disorders and highlights its utility as an additional tool in monitoring choroidal involvement in ocular inflammation. In an original article, “The Effects of Intravitreal Bevacizumab in Infectious and Noninfectious Uveitic Macular Edema,” H. Al-Dhibi et al. have reported the efficacy of intravitreal bevacizumab injections in the management of uveitic macular edema (UME) associated with both infectious and noninfectious uveitides. Importantly, intravitreal bevacizumab was found to induce remission of UME with no immunosuppressive effect against infectious agents. E. D'Ambrosio et al. in “Management of Uveitis-Related Choroidal Neovascularization: From the Pathogenesis to the Therapy” have reviewed the pathogenesis as well as management strategies for inflammatory choroidal neovascular membranes. L. Talat et al. in “Ischemic Retinal Vasculitis and Its Management” have reviewed the current options in the treatment of ischemic retinal vasculitis including the role of conventional as well as newer biological agents in the management of this challenging entity. Lastly, in a review by J. R. Maya et al., “Emerging Therapies for Noninfectious Uveitis: What May Be Coming to the Clinics,” the authors have reviewed all the emerging therapies for the management of noninfectious uveitis addressing the curiosity and concerns about the newer therapies that shall be coming to the clinics.

We sincerely hope that the readers will find these well-selected manuscripts of interest and obtain useful information to get an update on the management of uveitis.

*Vishali Gupta* *Vishali Gupta* *Quan Dong Nguyen* *Quan Dong Nguyen* *Manfred Zierhut* *Manfred Zierhut* *Ilknur Tugal-Tutkun* *Ilknur Tugal-Tutkun*

